# *Drosophila* Voltage-Gated Calcium Channel α1-Subunits Regulate Cardiac Function in the Aging Heart

**DOI:** 10.1038/s41598-018-25195-0

**Published:** 2018-05-02

**Authors:** Alexander Lam, Priyanka Karekar, Kajol Shah, Girija Hariharan, Michelle Fleyshman, Harmehak Kaur, Harpreet Singh, Shubha Gururaja Rao

**Affiliations:** 1 Department of Pharmacology and Physiology, Drexel University College of Medicine, Philadelphia, PA 19102 USA; 2 Division of Cardiology, Department of Medicine, Drexel University College of Medicine, Philadelphia, PA 19102 USA

## Abstract

Ion channels maintain numerous physiological functions and regulate signaling pathways. They are the key targets for cellular reactive oxygen species (ROS), acting as signaling switches between ROS and ionic homeostasis. We have carried out a paraquat (PQ) screen in *Drosophila* to identify ion channels regulating the ROS handling and survival in *Drosophila melanogaster*. Our screen has revealed that α1-subunits (D-type, T-type, and cacophony) of voltage-gated calcium channels (VGCCs) handle PQ-mediated ROS stress differentially in a gender-based manner. Since ROS are also involved in determining the lifespan, we discovered that the absence of T-type and cacophony decreased the lifespan while the absence of D-type maintained a similar lifespan to that of the wild-type strain. VGCCs are also responsible for electrical signaling in cardiac cells. The cardiac function of each mutant was evaluated through optical coherence tomography (OCT), which revealed that α1-subunits of VGCCs are essential in maintaining cardiac rhythmicity and cardiac function in an age-dependent manner. Our results establish specific roles of α1-subunits of VGCCs in the functioning of the aging heart.

## Introduction

Cellular Ca^2+^ signaling pathways interact with ion channels and are regulated by other cellular signaling systems such as reactive oxygen species (ROS)^1–4^. ROS is one of the major players in cellular growth, senescence, and death^5^. Increasing evidence suggests an intricate relation between Ca^2+^ and ROS where both signaling mechanisms finely modulate cellular functions^2^. Hence, dysfunction or dysregulation of any of these mechanisms could contribute to pathophysiological conditions such as neurodegenerative diseases and cardiac dysfunction. ROS have also been implicated in accelerated aging and old age, where severity, as well as susceptibility to neuronal and cardiac diseases, increases by many folds. Ion channels modulated by ROS are known to regulate cardiac function by controlling rhythmicity and contractility^6^. The α1-subunits of voltage-gated calcium channels (VGCCs) are the direct targets of ROS and their channel activities and their expression is regulated by ROS^7,8^. The thiol redox state of the channel protein is an important determinant of channel function, hence governing cellular fate. Given Ca^2+^ promotes ATP production and oxidative phosphorylation in the mitochondria, increase in Ca^2+^ results in increased respiratory chain electron leakage and ROS levels^9,10^. However, the direct role of VGCCs in ROS production and its physiological consequences are not known.

VGCCs mediated depolarization-induced Ca^2+^ influx influences a wide array of cellular and physiological functions. Ca^2+^ influx through VGCCs serves as a second messenger of electrical signaling; for example, in cardiac and smooth muscle cells, these channels initiate cellular contractions, in endocrine cells, they induce hormone secretion, and in neurons, they are the key players in synaptic transmission^11^. The α1-subunit of VGCCs has distinct biophysical properties, which differ from other Ca^2+^ channels. In mammals, there are several genes encoding for the α1-subunits. The identification of physiological roles of VGCCs in mammals is impeded by the potential compensation by products of different genes encoding for each of the α1-subunits and the lack of specific pharmacological inhibitors^12^. In contrast, *Drosophila melanogaster* (*Dm*) possesses one α1 subunit gene in each family. α1D [Ca_v_1, ~50% identical to human orthologs CACNA1C (Ca_v_1.2)/CACNA1D Ca_v_1.3], Cac [Ca_v_2, 43% identical to human ortholog CACNA1B (Ca_v_2.2)], and α1T [Ca_v_3, 34% percent identical to human ortholog CACNA1 (Ca_v_3.3)], hence providing genetic tools for imploring their physiological roles^13–16^. *Dm* Ca^2+^ channels are repeatedly implicated in Ca^2+^ currents responsible for the release of neurotransmitters in a wide array of excitatory neurons^17^ and in the modulation of sleep^18^. Therefore, we investigated the physiological role of α1-subunits of VGCCs at the organism level using *Drosophila* VGCC mutants.

In this study, we analyzed *Drosophila* ion channel mutants for their susceptibility to paraquat (PQ, 1,1′-dimethyl-4,4′-bipyridinium dichloride), an oxidative stressor. PQ is a widely used ROS inducer, which results in the rapid death of wild-type *Drosophila*. Our results indicate different ion channels showing different degrees of ROS handling and the outcome is also influenced by gender. Interestingly, we observed VGCCs showing differential responses to PQ-induced ROS handling compared to wild types. Given the interplay between calcium and ROS signaling, and ROS being known to play a role in determining the lifespan, we investigated the roles of VGCCs in the lifespan of *Drosophila*, using mutant flies for α1D (Ca_v_1), Cac (Ca_v_2), and α1T (Ca_v_3) ion channels. α1-subunits of VGCC family form the principal channel protein with voltage sensing domains, and the conduction pore with selectivity filter and activation gates of these subunits is enough to reconstitute functional channels^19^. To determine the amount of ROS generated by mitochondria of mutant VGCC flies, we measured ROS-generation, which indicated that the absence of VGCCs decreased ROS generation. Since VGCCs are known to participate in heart rate, rhythm, and function, we also determined the role of individual genes in cardiac function using state of the art optical coherence tomography (OCT). Our results for the first time demonstrate a direct role for each of the VGCCs in cardiac function of aging flies.

## Materials and Methodology

### Fly stocks

The fly stocks were obtained from Bloomington *Drosophila* Stock Center (Bloomington, IN) and incubated at 25 °C on jazz mix media. The wild-type (wt) used was the *Canton-S* (*Canton-S;* FBsn0000274) strain throughout all the experiments. The additional strains include T-type Ca^2+^ (*w*^1118^ *PBac*[*WHr*]*Ca-α*1*T*^*del*^*; CG*1*5899*), D-type Ca^2+^ (*b*^*1*^ *Ca-α1D*^*X7*^ *pr*^1^ *cn*^1^ *wx*^*wxt*^ *bw*^1^*/CyO; CG4894*), Cacophony (*cac*^*H18*^*; CG43368*);, DmNav (*para*^*ST76*^*; CG9907*), GluR IIA (*y*^*1*^ *w*^*67c23*^*; GluRIIA*^*MB01746*^*; CG6992*), Ir64a(*w*^1118^*; Ir64a*^*MB05283*^*; CG*1*0633*), HisCl1 (*st*^1^ *HisCl*1^*T2*^ *e*^*s*^*, CG*1*4723*), HisCl2 (*ort*^1^*; CG7411*), Hk (*Hk*^1^*; CG43388*), na (*na*^1^*/C(1)DX,y*^*1*^ *f*^*1*^*; CG1517*), nAchR alpha1 (*y*^*1*^ *w*^*67c23*^*; P*[*EPgy2*]*nAChRα1*^*EY09706*^*; CG5610*), nAchR alpha5 (*w*^1118^*; PBac*[*WH*]*nAChRα5*^*f00872*^*/CyO; CG32975*), NMDA receptor1 (*P*[*PZ*]*Itp-r83A*^05616^ *Nmdar1*^05616^ *ry*^506^*/TM3, ry*^*RK*^ *Sb*^1^ *Ser*^1^*; CG2902*), Trpml (w^*^; PBac[*GAL4D,EYFP*] *Trpml*^*PL00182*^ *P*[*FRT(w*^*hs*^)]*2AP*[*neoFRT*]*82B; CG8743*), Trp A1 (*w*^1118^*; TI*[*TI*]*TrpA1*^1^; CG5751), NaCP 60E (*w*^1118^*; P*[*EP*]*NaCP60E*^*EP348*^ *RpL41*^*EP348*^*/CyO; CG34405)*, NompC[h25] (*nompC*^*h25*^*/SM6b; CG11020*), Ryanodine receptor (RyR) 44F (*RyR*^*Q3878X*^*/CyO; CG10844*), and Shaker cognate 1 (*w*^1118^*; PBac*[*WH*]*Shal*^*f00495*^*/TM6B*, *Tb*^1^; *CG9262*).

### Paraquat experiments

Flies were starved for 2 hours in 0.5% (*w/v*) agarose and transferred into vials containing fiberglass filter papers with 5% (*w/v*) sucrose and 20 mM PQ^20–23^. The number of dead flies was recorded every 12 hours until all the flies died.

### Lifespan studies

The flies were collected once they were adults and contained in vials (10–15 flies in each vial, n = 3). The flies were incubated at 25 °C on jazz mix media. The number of deaths was recorded every 3 days until all the flies died.

### Geotaxis

A 5-centimeter mark was made in a vial, and the number of flies that crossed the mark in 18 seconds after tapping them to the bottom of the vial was recorded (5 flies each separated based on gender were tested three times).

### Mitochondrial Isolation

Mitochondria were isolated from 1–5 day old flies at 4 °C. About 50 mg of flies were homogenized in 2.0 mL isolation buffer A [70 mM sucrose, 210 mM Mannitol, 1 mM EDTA, 50 mM Tris HCl, pH 7.4] using a Potter-Elveiem homogenizer, according to the published protocol^24^. The homogenate was centrifuged at 2500 × g for 5 minutes, the supernatant was transferred to a 1.7 mL Eppendorf tube, and centrifuged at 12,000 × g for 10 minutes. The supernatant was then discarded, the pellet was resuspended in 100 μL isolation buffer B [70 mM sucrose, 210 mM mannitol, 0.1 mM EDTA, 50 mM Tris HCl, pH 7.4], and centrifuged at 12,000 × g for 5 minutes. Supernatant was again discarded, pellet was resuspended in 100 μL ROS buffer [250 mM sucrose, 1 mM EGTA, 1 mM EDTA, 0.15% (*w*/*v*) BSA, 20 mM Tris HCl, pH 7.4], and centrifuged at 12,000 × g for 5 minutes. The supernatant was discarded and the pellet was resuspended in 55 μL ROS buffer.

### Reactive Oxygen Species Measurement

ROS generation was detected by amplex red (Life Technologies) at 25 °C, using fluorescence spectrophotometer (Hitachi F-2710) described previously^4,15,16,25^. 5 μg horseradish peroxidase (Sigma-Aldrich) was added to the ROS buffer in a stirred measuring cuvette, and the baseline fluorescence was measured (excitation at 560 nm and emission at 590 nm) for 5 minutes. After 60 seconds, 10 μM amplex red was added to the cuvette, 60 seconds later, 25 μL of isolated mitochondria sample was added, followed by 3 μM succinate (Sigma-Aldrich) after 90 seconds. Fluorescence was monitored continuously for 45 minutes at 4 seconds resolution, and the rate, as well as total ROS production, were calculated.

### Cardiac function analysis

The wild-type and Ca^2+^ α1 subunit mutants (D-type, T-type, Cac) were selected at week one, three, and six to evaluate their cardiac function as they age, with the body length in the range around 2.5 to 3.0 mm. To image the flies, they were first immobilized using grease on slides. The flies were exposed to continuous CO_2_ for 30 seconds to 1 minute; the flies were placed on a thin layer of high vacuum grease (DOW Corning, Michigan, U.S.A.) with their dorsal side facing towards the optical coherence tomography (OCT) scope. The flies were given 20 minutes to awaken and placed under the OCT system.

TELESTO-II OCT system (Thorlabs, Munich, Germany) with a nominal wavelength of 1310 nm was used to obtain two-dimensional B-mode and two-dimensional M-mode traces. The traces were recorded through ThorImage OCT 4.4.6.0 software. The two-dimensional B-mode images had an axial scan (A-scan) line rate of 76 kHz, and the two-dimensional M-mode images had an A-scan line rate of 5.5 kHz. The cardiac tube was located by focusing the infrared beam at the abdomen of the fly and two-dimensional B-mode images were acquired. The system was then switched to two-dimensional M-mode to continuously record traces of the cardiac cycle.

The recorded two-dimensional M-mode OCT traces were stored and analyzed to quantify the end diastolic diameter (EDD), end systolic diameter (ESD) through the ThorImage software. The EDD and ESD is the distance between the superior and inferior sides of the heart walls in diastolic and systolic periods during normal cardiac cycles of each fly. The averages were taken for the EDD and ESD dimensions of each fly, and these dimensions were used to calculate the fractional shortening defined as [(EDD − ESD)/ESD] × 100. The 1.83 second M-mode images were used to calculate the heart rate during a normal cardiac rhythm. The cardiac parameters were averaged from a minimum of three cardiac cycles.

Each arrhythmic event was characterized as a cardiac contraction that had a diastolic interval that spanned at least twice the interval of a normal diastolic interval. Arrhythmic index^26^ was calculated by quantifying the number of arrhythmic events that occurred in the heart cycle and normalized to the heart rate of each individual fly.

### ROS Staining

Flies were dissected to expose the cardiac tube for reactive oxygen species (ROS) staining. Prior to dissection, the flies were immobilized after continuously exposing them to CO_2_ for 1 minute. The methodology of dissecting the adult fly was described previously^27^ to maintain a semi-intact beating heart before staining. After exposing the cardiac tube, the entire fly was submerged in 1:1000 dihydroethidium (DHE, Molecular Probes) in phosphate buffered saline (PBS) for 5 minutes. The DHE was washed with PBS two times, then fixed with 4.0% (*w/v)* paraformaldehyde for 3 minutes. The paraformaldehyde was washed with PBS two times, and the fly was mounted on a microscope slide. The cardiac tube was imaged using an Olympus confocal microscope [IX81] with a 60X objective lens [NA 1.42] at 568 nm within 30 mins to image ROS.

### Statistics

Data are shown as the average ± standard error mean. The statistical analysis was performed using a two-tailed t-test for single comparisons. Group comparisons were evaluated using one-way ANOVA with a post-hoc Tukey test (at least 3 trials with a minimum of 4 flies used in each trial).

## Results

### Ion Channel Screening for Oxidative Stress

Ion homeostasis is critical for cellular physiology such as proliferation, migration, cytokine secretion, apoptosis and cell death. Several cellular and extracellular factors regulate ion channels. Ion channels responsible for transportation of Ca^2+^ not only control ROS-generating enzymes and processes but also are regulated by cellular ROS^28,29^. Though channels are tightly modulated by ROS, the absence of ion channels affecting the susceptibility towards oxidative stress is not completely understood. To characterize the role of ion channels in handling oxidative stress, we measured survival of *Drosophila* ion channel mutants on the oxidative stress-inducing agent, PQ, a drug commonly used in fruit fly research^20–23^. PQ is shown to affect mitochondria in several organisms by altering mitochondrial function as well as mtDNA^21,30^. Routinely, gender is associated with differences in oxidative stress and it is believed that under physiological conditions females are less susceptible to oxidative stress^31^. To understand the role of ion channels in oxidative stress and the direct relation with gender, we screened males and females, separately.

**Figure 1:**
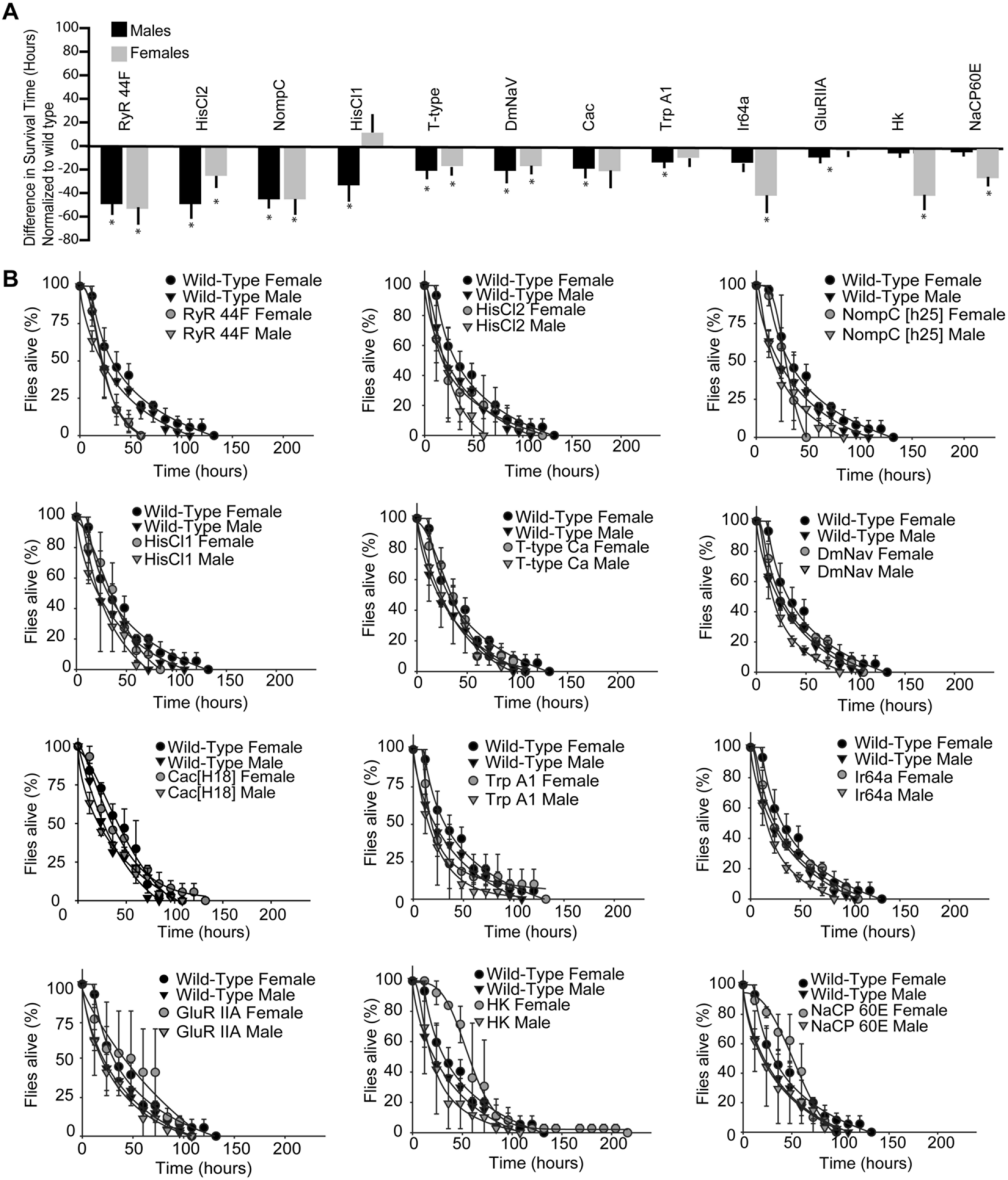
*Drosophila* lacking ion channel mutants with high sensitivity to PQ. (**A**) Bar graphs representing the survival of *Drosophila* ion channel mutants normalized to male wild-type (*Canton-S*) flies. Black bars represent males and gray bar represent females. Two way ANOVA was performed and statistical significance (p < 0.05) are indicated by an asterisk. HisCl1, Trp A1 and GlurRIIA female mutant flies, and Ir64a, Hk and NaCP60E mutant male flies were not sensitive to PQ. (**B**) Line graphs represent the survival of ion channel mutant flies subjected to 20 mM PQ.

**Figure 2:**
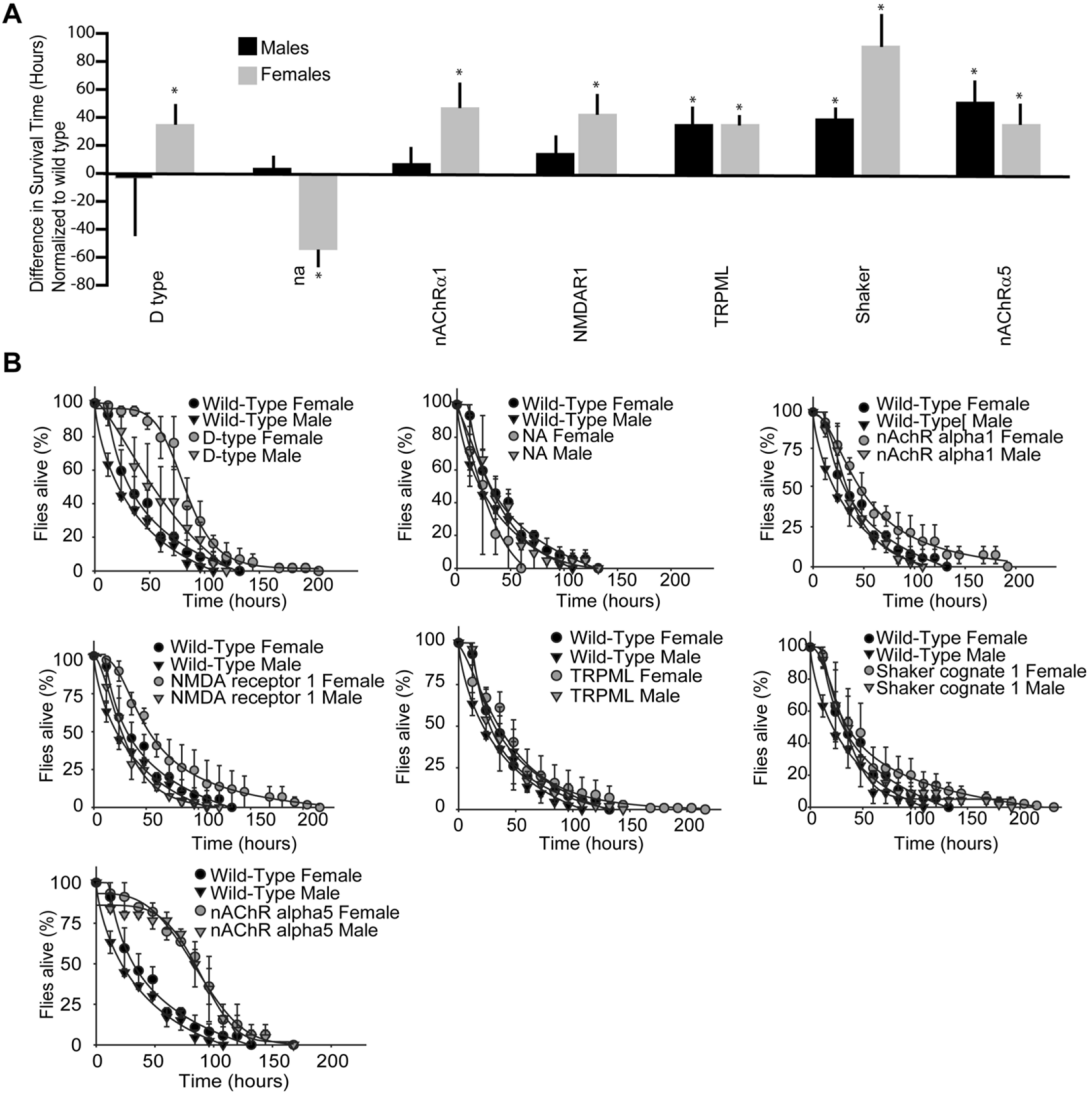
*Drosophila* lacking ion channel mutants resistant to PQ treatment. (**A**) Bar graphs representing PQ resistant *Drosophila* ion channel mutants normalized to male wild-type (*Canton-S*) flies. Black bars represent males and gray bar represent females. Two way ANOVA was performed and statistical significance (p < 0.05) are indicated by an asterisk. D type Ca^2+^ channels, Na, nAChRα1 and NMDAR1 male mutant flies were not resistant to PQ and were found with sensitivity similar to wt flies. (**B**) Line graphs represent the survival of ion channel mutants subjected to 20 mM PQ.

We carried the oxidative stress study by feeding flies with PQ for a minimum of 10 flies in each group. Survival of different ion channel mutants was normalized for male wild-type flies (Fig. 1A). Even though females tend to show a better handling of oxidative stress, there were differences between survival of males and females (Fig. 1A,B, black circles and triangles) among various mutants. Ion channel mutants for RyR 44F, HisCl2, NompC, HisCl1, T-type Ca^2+^, DmNaV, Cac, TrpA1, Ir64a, GluRIIA, Hk, and NaCP60E performed worse than wild-type flies (Fig. 1B). We observed significant differences between males and females for HisCl2, HisCl1, Ir64a, Hk and NaCP60E (Fig. 1A,B). More importantly, female HisCl1 mutants (Fig. 1A) and Na mutants (Fig. 2A) handled stress better HisCl1 and Na mutant male flies, respectively. *Drosophila* mutants for nACHRα1, NMDAR1, TRPML, Shaker and nACHRα5 channels were significantly resistant to oxidative stress as compared to the wild-type flies (Fig. 2A,B). In oxidative stress-resistant flies, female mutants for D-type Ca^2+^, nACHRα1, NMDAR1, and Shaker handled stress better than their male counterparts (Fig. 2A,B). No gender biased for stress handling was observed for RyR44F, NompC, T-type Ca^2+^, DmNaV, TrpA1, GluRIIA, TRPML, and nACHRα5 channel mutants (Fig. 2A,B). Some of these stocks were homozygous lethal, however, the presence of balancer chromosomes did not affect the paraquat sensitivity of the flies (Supplemental Fig. 1). Our results showed that absence ion channels regulate the ROS stress handling by flies. These findings showed for the first time that the gender bias for handling stress involves ion channels and possibly other signaling pathways. Amongst these channels, we chose to further study the physiological roles of VGCCs as they showed the most dramatic effects.

### Lifespan

In our PQ screening, one of the most significant classes of ion channels displaying variability in survivability was VGCCs. Even though VGCCs result in Ca^2+^ transport and belong to the same family, the outcome of their absence was at extreme ends. In mammals, the role of VGCCs in physiology is not well-defined due to compensation by other VGCCs. In mammalian coronary arteries, it is known that VGCCs-mediated currents and gene expression decrease with aging, however, the role of VGCCs in the regulation of lifespan is not known. In order to understand the role of VGCCs in *Drosophila*, we performed the survivability assay. Our PQ screen showed a gender bias in handling the oxidative stress by some of the VGCCs (Figs 1 and 2), hence we performed the lifespan assay on males and females separately. For comparison and controls, we have used the Ca-S strain wild-type flies.

**Figure 3:**
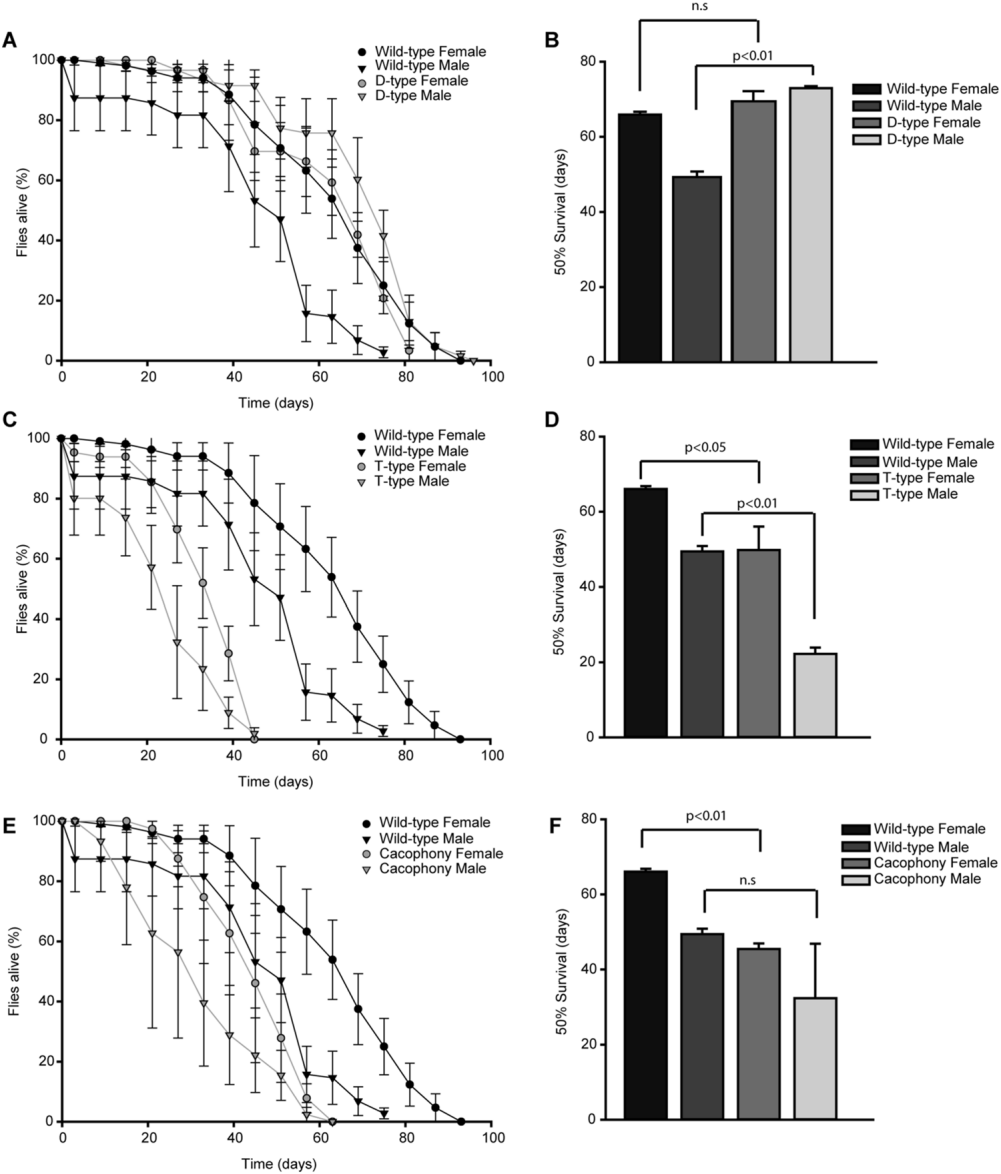
Role of VGCCs in the survival of *Drosophila. Drosophila* α1 mutants (D-type Ca^2+^ channels, **A**), (T-type Ca^2+^ channels, **C**) and (Cac, **E**) and wild-type flies were scored every 3 days. Bar graphs representing 50% survival of *Drosophila* mutants for (D-type Ca^2+^ channels, **B**), (T-type Ca^2+^ channels, **D**) and (Cac, **F**). D-type Ca^2+^ channel mutant males showed increased lifespan as compared to the wild-type (**A**,**B**). T-type Ca^2+^ channel mutants showed decreased lifespan for both genders as compared to the wild-type (**C**,**D**). There was no significant difference between Cac males and females (**E**,**F**).

The absence of D-type Ca^2+^ channels (D-type) showed that lifespan of female flies was similar to wild-type flies but males showed a significant increase in lifespan. The 50% survival for D-type was 73 ± 1 and 70 ± 2 days for males and females, respectively. The increase in D-type lifespan was 20% higher than wild-type in males (Fig. 3A,B). However, the absence of T-type Ca^2+^ channels (T-type) showed a significant decrease in survival. The 50% survival of T-type was 22 ± 2 and 50 ± 6 days for males and females, respectively (Fig. 3C,D). The decrease in lifespan for T-type flies was 25% and 15% for males and females, respectively as compared to the wild-type males and females. Absence of cacophony channels (Cac) showed a similar trend as a T-type for females; the 50% survival of Cac mutant flies was 44 ± 2 days for females (Fig. 3E,F). The decrease in lifespan of Cac was 15% and 20% for males and females, respectively, as compared to the wild-type males and females. Our results surprisingly indicated that the expression of VGCCs (T-type and Cac) is important for the survival of flies, however, the absence of D-type increases the lifespan in males.

### Negative geotaxis

**Figure 4:**
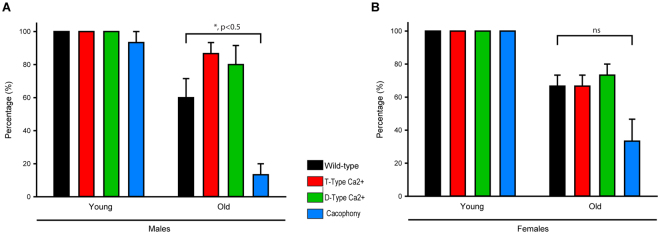
Role of α1 channels in locomotion. The locomotive ability of wild-type and α1 mutants were characterized through negative geotaxis, by the percentage of flies that were able to move a certain distance up the vial. (**A**) Male-specific negative geotaxis assessment in relation to young and old flies. (**B**) Female specific negative geotaxis assessment in relation to young and old flies. Significant differences were compared between young and old flies of their respective genders (n = 3 trials with a minimum of 5 flies each).

Locomotor impairment associated with aging in humans is a hallmark of old age. In *Drosophila*, age-related locomotor impairment is well characterized. Since we observed differences in the lifespan of VGCCs null mutants, we carried out negative geotaxis assays on all the VGCCs in both genders. In young male and female flies, there were no significant differences observed for negative geotaxis. However, in older flies, all the mutants and wild-type flies showed reduced locomotion. In old males, Cac mutants locomotion was significantly impaired as compared to wild-type flies but no difference was observed in D-type, T-type or wild-type flies (Fig. 4A). In old females, no change was observed between D-type, T-type, Cac, or wild-type flies (Fig. 4B). These data demonstrate that there is gender bias on locomotor impairment associated with old age in Cac mutants.

### Reactive Oxygen Species

**Figure 5:**
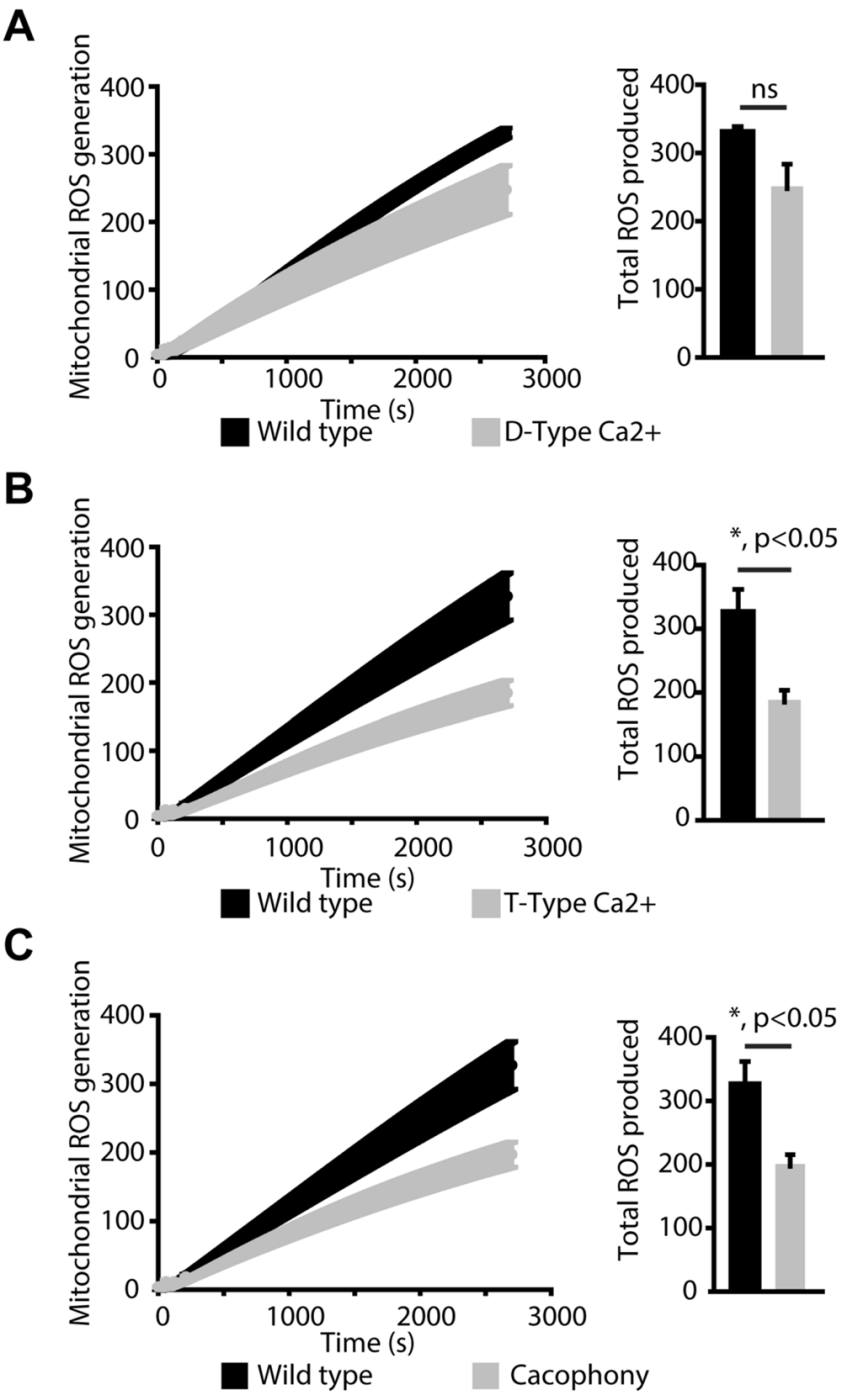
Mitochondrial ROS production. ROS generated by isolated mitochondria was measured using amplex red and succinate as a substrate. (**A**) ROS production by mitochondria isolated from the D-type Ca^2+^ channel (grey) and wild-type (black) normalized to the protein concentration. Bar graphs represent quantification of A. (**B**) ROS production by mitochondria isolated from the T-type Ca^2+^ channel (grey) and wild-type (black) normalized to the protein concentration. Bar graphs represent quantification of B. (**C**) ROS production by mitochondria isolated from Cac channel (grey) and wild-type (black) normalized to the protein concentration. Bar graphs represent quantification of C.

ROS are known to regulate lifespan and are a key component of aging-related disorders. Since our results showed that PQ-mediated ROS generation has a specific impact on VGCCs (Figs 1 and 2) and there are significant differences in survival of flies under stress as well as under physiological conditions (Fig. 3), we tested mitochondrial ROS generation. The major source of ROS in cells is mitochondria, hence we isolated mitochondria from the whole flies to measure the role of VGCCs in mitochondrial ROS generation. ROS were measured from isolated mitochondria in presence of succinate as a substrate^4,25^. Each mutant fly was compared with the wild-type. As shown in Fig. 5A, even though the amount of ROS generated in D-type mutants was lower, we did not observe any significant differences as compared to the wild-type mitochondria. In contrast, T-type and Cac mutants showed a significant reduction in mitochondrial ROS production as compared to the wild-type flies (Fig. 5B,C). We also performed staining experiments using DHE, an indicator of ROS, in the cardiac tubes. We observed decreased levels of staining for ROS in all VGCC mutants (Supplemental Fig. 2) in agreement with decreased mitochondrial ROS generation (*c.f*. Fig. 5). Our results surprisingly demonstrate that even though a significant reduction in lifespan for T-type and Cac mutants were observed, there was no increase in ROS levels.

### Cardiac function

**Figure 6:**
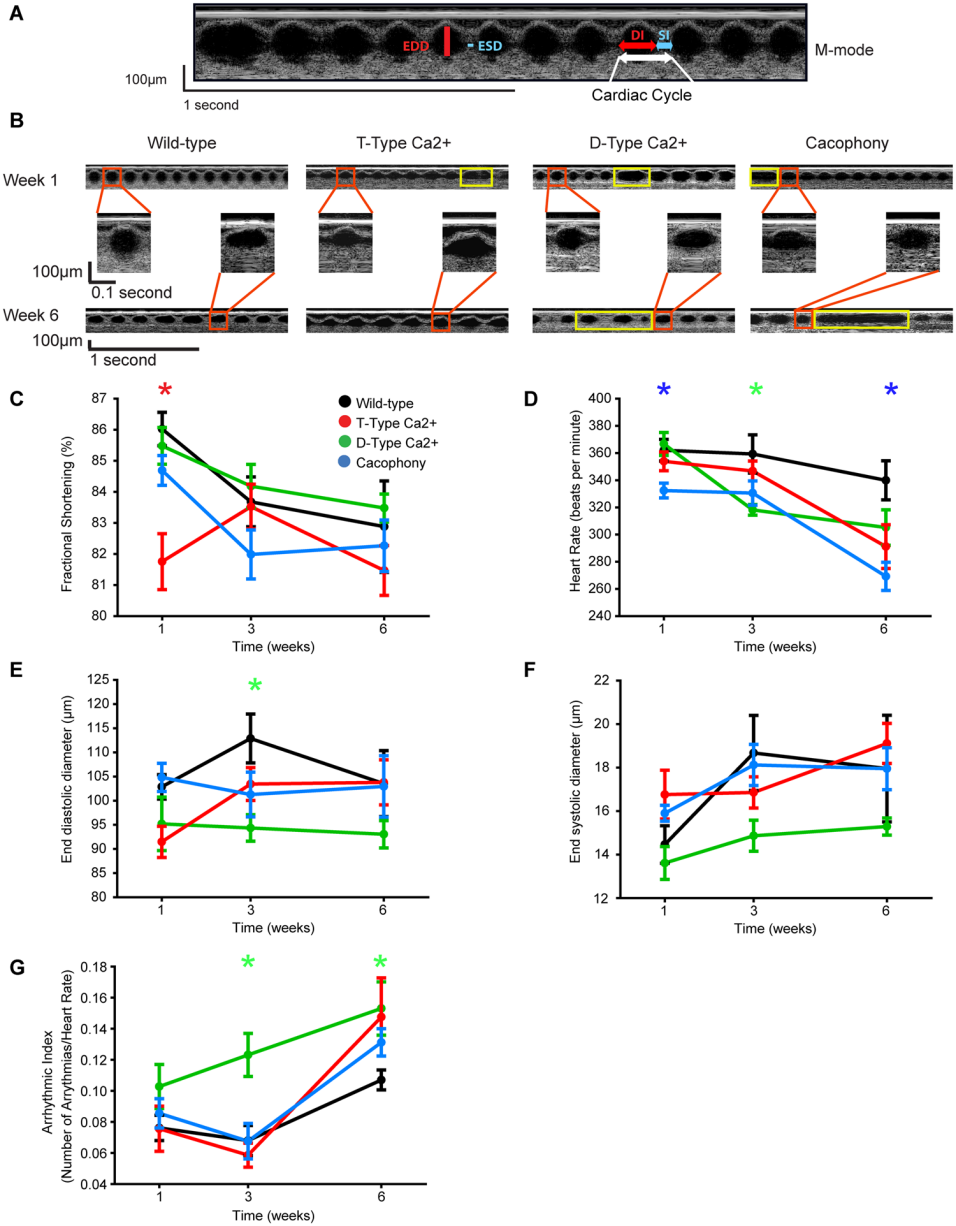
Cardiac function of *Drosophila* VGCCs. (**A**) Representative M-mode OCT traces defined the dimensions of the cardiac tube in a 1-week old wild-type (*Canton-S*) fly. The end diastolic diameter (EDD, red bar) and end systolic diameter (ESD, blue bar) were the cardiac dimensions used to quantify fractional shortening. The diastolic interval (DI, red arrow) and systolic interval (SI, blue arrow) define the cardiac period (white arrow). (**B**) Cardiac function was evaluated using M-mode traces of the cardiac tube in wild-type and α1 mutants in relation to age. Enlarged individual events are shown in the red boxes. The prominent arrhythmic events that occurred in the 1 week and 6-week old mutants are shown in the yellow boxes. The cardiac parameters include fractional shortening (**C**), heart rate (**D**), end diastolic diameter (**E**), end-systolic diameter (**F**) and arrhythmic Index (**G**). Significant differences between wild-type and mutants were determined by one-way ANOVA followed by a post-hoc Tukey test (*p < 0.05, n = 11 to 16).

The functional role of VGCCs in cardiac function is well characterized; however, the specific role of each VGCC is not yet deciphered. Over the last 15 years, VGCCs have been shown to contribute to cardiac pacemaking, atrioventricular conduction, and heart rate determination^32^. Since VGCCs null mutants showed susceptibility to ROS stress (Figs 1 and 2), and oxidative stress dysregulates Ca^2+^ metabolism, generally causing a rise intracellular Ca^2+,^ affecting the cardiac function, we tested whether the absence of VGCCs has a specific impact on cardiac function. We characterized the role of each of the VGCCs in cardiac function in the aging heart of *Drosophila* noninvasively by using OCT (Fig. 6A).

Cardiac tubes were imaged for a minimum of 60 seconds for wild-type and mutant flies (D-type, T-type and Cac) (Fig. 6B). We observed no changes in the fractional shortening (Fig. 6C), heart rate (Fig. 6D), end diastolic (Fig. 6E) and systolic diameter (Fig. 6F) of wild-type flies with age. Surprisingly T-type showed a reduced fractional shortening of 82 ± 1% at week 1 as compared to the wild-type flies 86 ± 0.5% (n = 11 to 13 flies, Fig. 6C,E). At week 3, all the VGCCs showed a relatively smaller EDD as compared to wild-type (Fig. 6E). Since VGCCs are known to play a role in heart rate, we also calculated the heart rate through M-mode OCT traces. As shown in Fig. 6D, the heart rate of all the VGCCs decreased with age (week 1 vs week 3 and 6) as compared to the wild-type. For instance, week 6 aged wild-type flies showed a higher heart rate of 340 ± 14 beats per minute (bpm) compared to the heart rate in D-type, T-type, and Cac of 305 ± 13 bpm, 291 ± 16 bpm, and 269 ± 10bpm, respectively. In young week 1 flies, Cac showed a significant decrease in heart rate of 332 ± 5 beats per minute compared to the heart rate of 362 ± 8 beats per minute in wild-type (Fig. 6D). Out of all the VGCCs, only the Cac showed a significant decrease in heart rate in relation to wild-type from weeks 1 to 6.

Arrhythmias characterized by long diastolic intervals without well-defined systolic events and inconsistent cardiac rhythm (yellow box, Fig. 6B) were observed in all the VGCCs mutants. Arrhythmias were quantified as an arrhythmic index^26^, which is the ratio between the number of arrhythmic events in relation to the heart rate. The index does not change for wild-type, T-type, and Cac in week 3 but increased for D-type. The D-type also showed a significant increase in arrhythmic events in week 6 compared to wild-type (Fig. 6G).

We also observed that the cardiac dysfunction noted specifically in the D-type mutants have a gender bias. At week 1, only male mutants showed a significant decrease in EDD compared to male wild-type (Supplemental Fig. 3B,D). In addition, the male mutants showed a significant decrease in EDD at week 1 compared to their female counterparts (Supplemental Fig. 3D,E). Male mutants had an EDD of 77.8 ± 3.8 µm (n = 4 to 7) which were significantly shorter compared to 112.6 ± 4.1 µm seen in female mutants (Supplemental Fig. 3D,E).

## Discussion

Oxidative stress resulting from an imbalance between oxidation and antioxidant systems is associated with a variety of diseases including diabetes mellitus, hypertension, atherosclerosis, and cardiovascular dysfunction^33^. Under physiological and pathological conditions, ROS may be harmful or beneficial to specific cell types. Sulfhydryl groups of cysteine and methionine residues in ion channels can be targeted by ROS altering their biophysical properties and corresponding signaling pathways^34^. The absence of ion channels from specific signaling pathways could interrupt the vital signaling mechanism affecting cellular physiology. Oxidative stress affects males and females differentially^31^ and, in our results, we discovered that several ion channels contribute towards this gender basis. Ion channels for HisCl1, D-type Ca^2+^, nACHRα1, NMDAR1, and Shaker cognate 1 showed more susceptibility for males, whereas Ir64a, Hk, NaCP60E, and na showed more susceptibility for females. Importantly, RyR44F, HisCl2, NompC, Cac, T-type Ca^2+^, DmNaV, TrpA1, GluRIIA, TRPML, and nACHRα5 showed no gender-based bias. These results are significant as we have discovered that the gender differences in oxidative stress are directly related to specific ion channels. In pathophysiological conditions such as cardiovascular diseases, gender differences are remarkably prevalent^31^. Males are more prone to myocardial dysfunction and infarction at the relatively young age but females are more susceptible to cardiovascular diseases after menopause. One of the aspects of this gender-based bias could be attributed to the antioxidant properties of estrogen in females^31^.

ROS have been directly associated with Ca^2+^ homeostasis in cells and intracellular organelles. Intracellular Ca^2+^ channels located in endoplasmic reticulum and mitochondria are intrinsically coupled with cellular ROS. The role of VGCCs in ROS modulation and how these channels can play a role in ROS handling is not known. In our PQ screening, we have discovered that absence of different VGCCs can have an independent effect on the survivability of *Drosophila* (Figs 1 and 2). Since ROS levels are known to increase with age and VGCCs mutant flies showed different ROS handling capabilities, we further tested the lifespan of flies lacking α1D (Ca_v_1), Cac (Ca_v_2), and α1T (Ca_v_3) channels. It is extremely challenging to test the role of VGCCs in mammalian systems such as mice due to compensation by other subunits of VGCCs. In flies, there are three distinct genes encoding VGCCs and mutant flies can be used to test the role of each of these genes. There are several diseases and disorders associated with VGCCs affecting the survivability of patients, for example, hypokalemic periodic paralysis (HypoPP) resulting from missense mutations in Ca_v_1.1 affect the second decade of life^35^, and Timothy syndrome due to *de novo* gain of function mutation in the pore-forming Ca_v_1.2 causes lethal tachycardia and reduce life expectancy by 2.5 years^36^. Ca_v_1.3^−/−^ mice are more vulnerable to ventricular extrasystoles and atrial fibrillation but have a normal lifespan^37,38^. In *C. elegans*, screening of pharmacological agents showed that Ca^2+^ channels can be targeted for oxidative stress and manipulation of lifespan^39^. Our study for the first time showed a differential role of each gene responsible for VGCCs (Fig. 3). The expression of VGCCs (T-type and Cac) is vital for the survival of flies under physiological conditions. In contrast, the absence of D-type Ca^2+^ channel is beneficial for male flies as it increased the lifespan. This could be due to *Drosophila* males responding to mild stress better than females^40^. Certain signaling pathways are also known to influence lifespan with a gender bias such as insulin signaling pathway^41^. Thus, our results suggest that different VGCCs influence aging and lifespan differently perhaps due to their differential signaling mechanisms. Negative geotaxis routinely used in studies for determining the age-associated locomotor disorder revealed that expression of Cac is vital for maintaining critical locomotion during old age. In flies, Cac and D-type Ca^2+^ directly interact with BK_Ca_ channels (*slo*)^42^ and have a differential effect on homeostasis regulation of presynaptic transmitter release and postsynaptic quantal response^43^. Cac mutants, when expressed in *slo* mutants, results in severe reduction in the excitatory junctional potential size and drastically decrease vesicle release but Dmca1D had no clear physiological modification of *slo*-induced synaptic homeostasis^43^. Also, *slo* mutants are known for their locomotory and neurological defects^44,45^. These studies in agreement with our findings demonstrate that Cac is essential for aging-related physiology possibly via interactions with BK_Ca_ channels.

As discussed earlier, ROS is known to play a dominant role in the determination of lifespan and aging. Although our results revealed a reduction in lifespan in T-type and Cac mutants, their respective mitochondrial ROS levels were significantly lower than wild-type. In contrast, D-type mutants showed an improved lifespan but there was no difference in ROS generation, though there was a trend towards reduction in ROS. This could be attributed to the independent role of D-type channels either by interacting with certain proteins or by changing ionic homeostasis in specific cellular compartments. Our staining experiments also supported this trend where we saw a decrease in ROS levels in all VGCC mutants. This decrease in ROS is seen at an isolated mitochondrial level from the whole flies as well as specifically in the intact cardiac tubes. Although we cannot rule out systemic effect on the cardiac tubes, the reduction in ROS in the heart is significant. These findings are important as they show that ROS is not playing a role in decreasing or increasing the survivability of VGCC mutants. In *Drosophila*, reduction in ROS is presumed to be associated with increased lifespan; for example, in flies that overexpress enzymes that destroy ROS (catalase and superoxide dismutase)^46,47^. Flies with a mutation in the *methuselah* gene can handle PQ-mediated ROS stress and is known to survive ~35% longer than wild-type flies^48^. In contrast, the involvement of ROS in mammalian aging is not clear. Mice with mutations in certain ROS-degrading enzymes do not cause premature aging^49,50^ but mice with lack of p66^shc^ protein live one-third longer than their wild-type littermates as a lack of p66^shc^ protein in cells resulted in resistance to ROS stress^51^. Our results indicate that VGCCs mutants could have more genetic redundancy and other genes may be differentially-regulated to produce ROS-handling enzymes. VGCCs are responsible for cellular Ca^2+^ changes and hence in turn results in Ca^2+^ homeostasis in mitochondria. Ca^2+^ homeostasis is extremely important for mitochondrial bioenergetics and ROS. Whilst there is no evidence for the presence of VGCCs in mitochondria, they are shown to affect Ca^2+^ loading into the mitochondria and hence can influence the function of mitochondria^52^. We believe VGCCs can have a significant influence on cardiac mitochondria specifically, given heart is an organ-dependent on mitochondria to maintain its function.

VGCCs are expressed in all regions of the heart including pacemaker cells and the conduction system. Dysregulation of L-type Ca^2+^ channels (α1D) mediated currents in sinoatrial nodal cells (SAN) causes cardiac arrhythmia and in mammals both Ca_v_1.2 and Ca_v_1.3 mediate sinoatrial L-type currents. In mice, Ca_v_1.3 specifically impact pace making^53^ and in humans, a loss of function mutation results in sinoatrial node dysfunction and deafness^54^. Ca_v_1.3 also conducts L-type current in atrioventricular node (AVN) cells and mice lacking the gene display AV-node conductance disturbances^55^. Similar to α1D, α1T channels are expressed in SAN and AVN cells, and only in neonatal myocytes. Ca_v_3.2 channels do not play a significant role in the generation of pacemaker potentials and mice lacking Ca_v_3.2 channels have a normal heart rate without any arrhythmias. Mice lacking Ca_v_3.1 channels have decreased pacemaker activity and AV conduction compared to the wild-type mice. Unlike α1D and α1T, the functional role of Cac (Ca_v_2) expressing R-type currents is not well-established. In recent studies, Ca_v_2.3 was shown to be involved in maintaining the cardiac autonomous nervous system and in intrinsic rhythm propagation^56^. Since VGCCs in mammals are expressed by multiple genes and regulatory subunits, their individual roles in cardiovascular function are not facile to dissect.

Our results indicate that all VGCCs are required for maintaining the heart rate but expression of α1T is critical in older flies (6 weeks). The heart rate of 3 and 6-week old flies were lower for all VGCCs mutants. Flies lacking α1D and Cac showed arrhythmias in week 6, and arrhythmias were already present in these mutants since week 1.The cardiac rhythmicity declines in both the wild-type and mutant 6-week old flies, which is consistent with previous studies^26,57^. Several attempts have been made to measure direct cardiac function in mice lacking different genes responsible for expression of VGCCs. However, due to challenges associated with the survivability of mice, these studies are extremely difficult to carry out. Ca_v_1.2 null mutant mice are embryonic lethal^58^ and cardiac-specific deletion of one or two Ca_v_1.2 wild-type alleles revealed that less than 50% reduction of I_Ca_ is not tolerated, and results in heart failure and enhanced lethality^59^. Heterozygous mice were also susceptible to ventricular dilation and cardiac hypertrophy on pathological or physiological stress^59^. Using *Drosophila* mutants, we were able to characterize the functional role of α1D; we observed a decrease in end-diastolic diameters (EDD) throughout development (week 1–6). Surprisingly these effects were gender specific in α1D mutants; EDD was significantly reduced in week 1 old male flies compared to week 1 old young female flies. In females, even though the EDD was larger than wild-type at week 1, the EDD decreased with age. ESD was relatively constant in all age groups in female flies. Another interesting observation was a slight increase in EDD in wild-type flies at week 3 but not that prominent in Ca^2+^ channel mutants, specifically D-type. Our observations are in agreement with earlier published data in mice^60,61^ and human beings^62^ where an increase in EDD is reported with age. Lack of increase in EDD for Ca^2+^ channel mutants could point to abnormal cell development due to abnormal Ca^2+^ flux which is governed by VGCCs in cardiomyocytes.

T-type Ca^2+^ channels are expressed in both left and right ventricles in mammals. Studies in isolated cells and animal models of heart failure implicate α1T channels in cardiac arrhythmias but evidence for their role in cardiac contractile function is less compelling. The functional overlap between Ca_v_3.1 and Ca_v_3.2 and splice variation associated with them in the heart also makes it difficult to elucidate roles of α1T channels in cardiac function. For example, cardiac function and development are not altered in Ca_v_3.2^−/−^ mice but had consequences for the hypertrophy response. In Ca_v_3.1^−/−^ mice LV hypertrophy on trans aortic constriction was suppressed in absence of Ca_v_3.2 and not enhanced as anticipated^63,64^. Our results have implicated α1T channels in cardiac function in addition to the modulation of heart rate. At week 1, there were no differences observed in the heart rate of α1T mutants and wild-type flies; however, there was a significant reduction in fractional shortening.

Ca_v_2 (R-type channel, Cac) are relatively understudied in cardiac function. In mice lacking Ca_v_2.3, no differences were observed in heart rate or heart function^65^. However, a four-fold increase in arrhythmias was observed in Ca_v_2.3^−/−^ mice^65^. An autonomic block of Ca_v_2.3 demonstrated that the increase in heart rate could be ascribed to increased sympathetic tonus in null mutant mice probably due to the enhanced anxiety levels^66^. Autonomic block, however, did not completely ablate increased heart rate and coefficient of variance and did not affect ectopic atrial/AV escape rhythms and QRS abnormalities, implicating Ca_v_2.3 in cardiac function^67,68^. In our experiments, we have observed the role of Cac in the regulation of heart rate in all age groups. At an older age, flies recovered EDD but their heart rate was significantly lower compared to wild-type.

Our results evidently implicate VGCCs in cardiac function. VGCCs are vital in maintaining heart rate and with age, the absence of these channels can have a detrimental effect on cardiac rhythm as well as contractility. Surprisingly all the VGCCs in flies showed a shorter EDD at week 3 but Cac and T-type flies recovered possibly due to compensation by other Ca^2+^ channels such as D-type Ca^2+^ channels. However, D-type flies could not recover revealing a detrimental role of the absence of α1D in cardiac dysfunction. These inferences are in agreement with earlier findings^61^ that lack of Ca^2+^ influx from Ca_v_1 channels leads to diastolic dysfunction. During aging, action potential, transient increase in cytosolic Ca^2+^, and rate of contraction is prolonged which consequently prolong systole and diastole events in the heart^69,70^, consistent with the lower maximal heart rate seen in older individuals even during exercise. Since we focused on Ca^2+^ channel mutants, our results could implicate these channels in age-associated prolong systole and diastole events of the heart. However, further work is required to tease out the role of individual VGCCs in aging hearts. Taken together our work has established the role of VGCCs in the lifespan of flies and cardiac function in an age-dependent manner.

## Electronic supplementary material

Supplementary Information
