# Supplementary material for: Drosophila Voltage-Gated Calcium Channel α1-Subunits Regulate Cardiac Function in the Aging Heart
Source: Sci Rep. 2018 May 2;8:6910. doi: 10.1038/s41598-018-25195-0 (PMC5932002; doi:10.1038/s41598-018-25195-0)

***Drosophila* Voltage-Gated Calcium Channel α1-Subunits Regulate Cardiac Function in the Aging Heart**

Alexander Lam^1^, Priyanka Karekar^1^, Kajol Shah^1^, Girija Hariharan^1^, Michelle Fleyshman^1^, Harmehak Kaur^1^, Harpreet Singh^1,2,^* and Shubha Gururaja Rao^1,^*

**Supplementary Figure 1 Wild-type and heterozygous *Drosophila* with balancer show similar sensitivity to PQ. A**. Male wild-type (blue), heterozygous with second chromosome balancer (red), and third chromosome balancer (green) *Drosophila* show similar sensitivity to PQ. **B.** Female wild-type (blue), heterozygous with second chromosome balancer (red), and third chromosome balancer (green) *Drosophila* also show similar sensitivity to PQ.

**
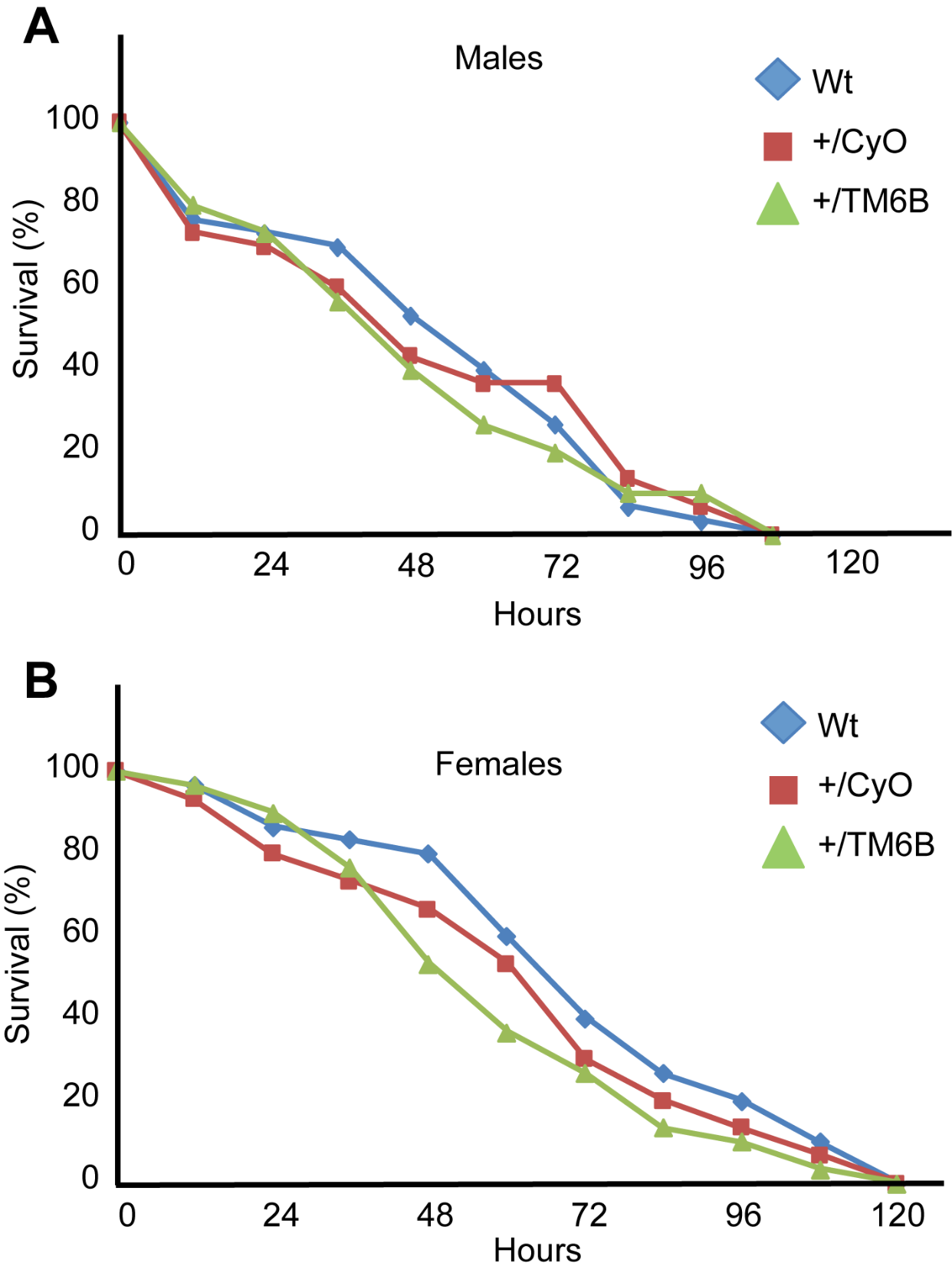
**

**Supplementary Figure 2 ROS staining of the cardiac tubes in wild-type and VGCC mutants.** Dihydroethidium (DHE) staining of the dissected cardiac tubes (highlighted with an outline and white arrow) in 3-week old wild-type and VGCC mutants. The amount of ROS was lower in all VGCC mutants as compared to the wild-type flies. Pericardial cells and fat tissues (outside the highlighted area) show similar levels of ROS.

**
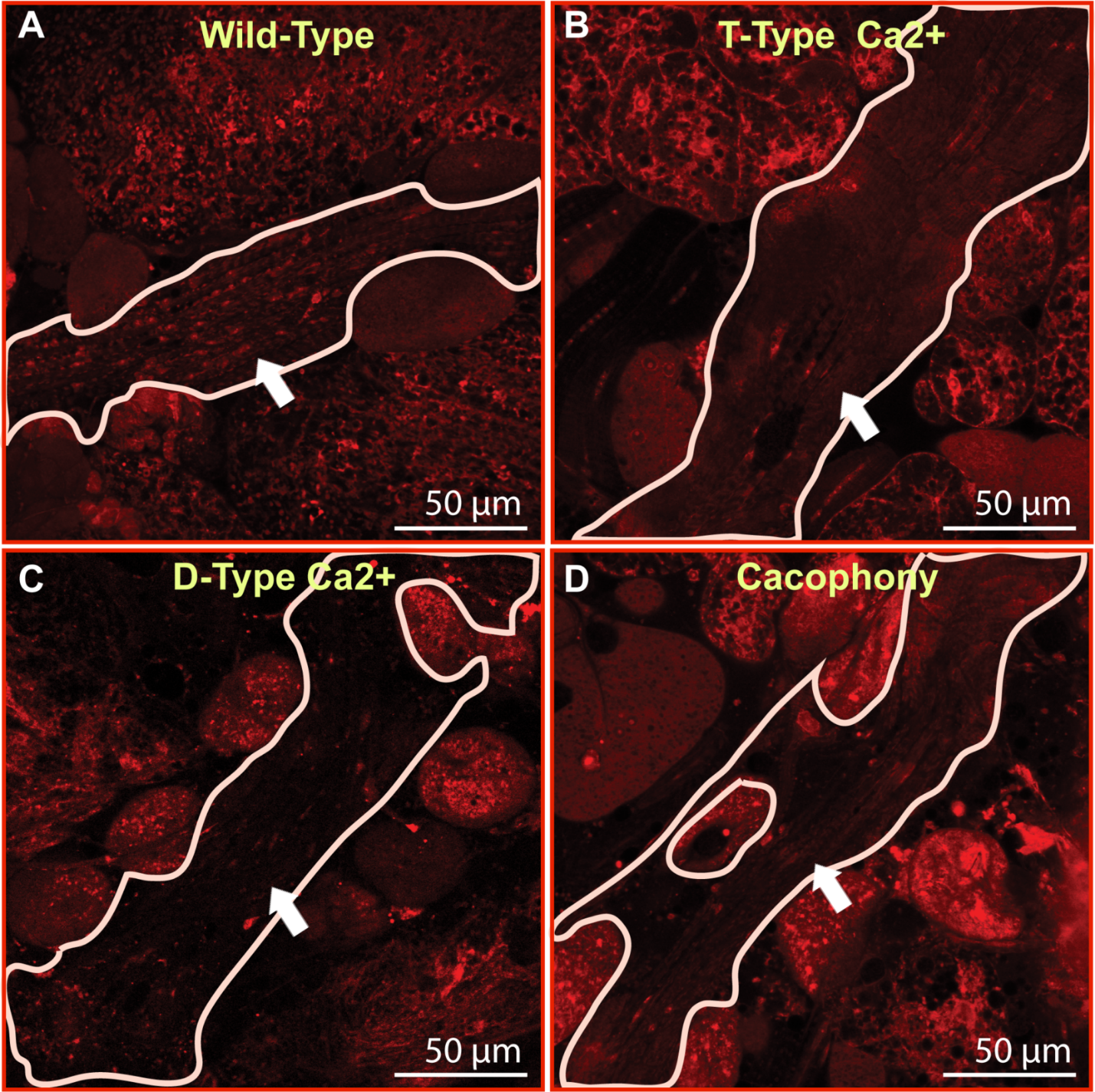
**

**Supplementary Figure 3 Gender bias in cardiac parameters of D-type Ca^2+^ channel mutants. A-D.** D-type Ca^2+^ channel mutants showed a gender bias in cardiac dimensions (EDD) only in week one. The cardiac function of D-type Ca^2+^ channel mutants was further analyzed and compared to wild-type in relation to age and gender. Cardiac events are highlighted with a red line and enlarged in black boxes. The cardiac function analysis includes fractional shortening (**E**), heart rate (**F**), end diastolic diameter (**G**), and end-systolic diameter (**H**). The significant differences of each mutant were compared to wild-type (* = p<0.05, n=4 to 7).


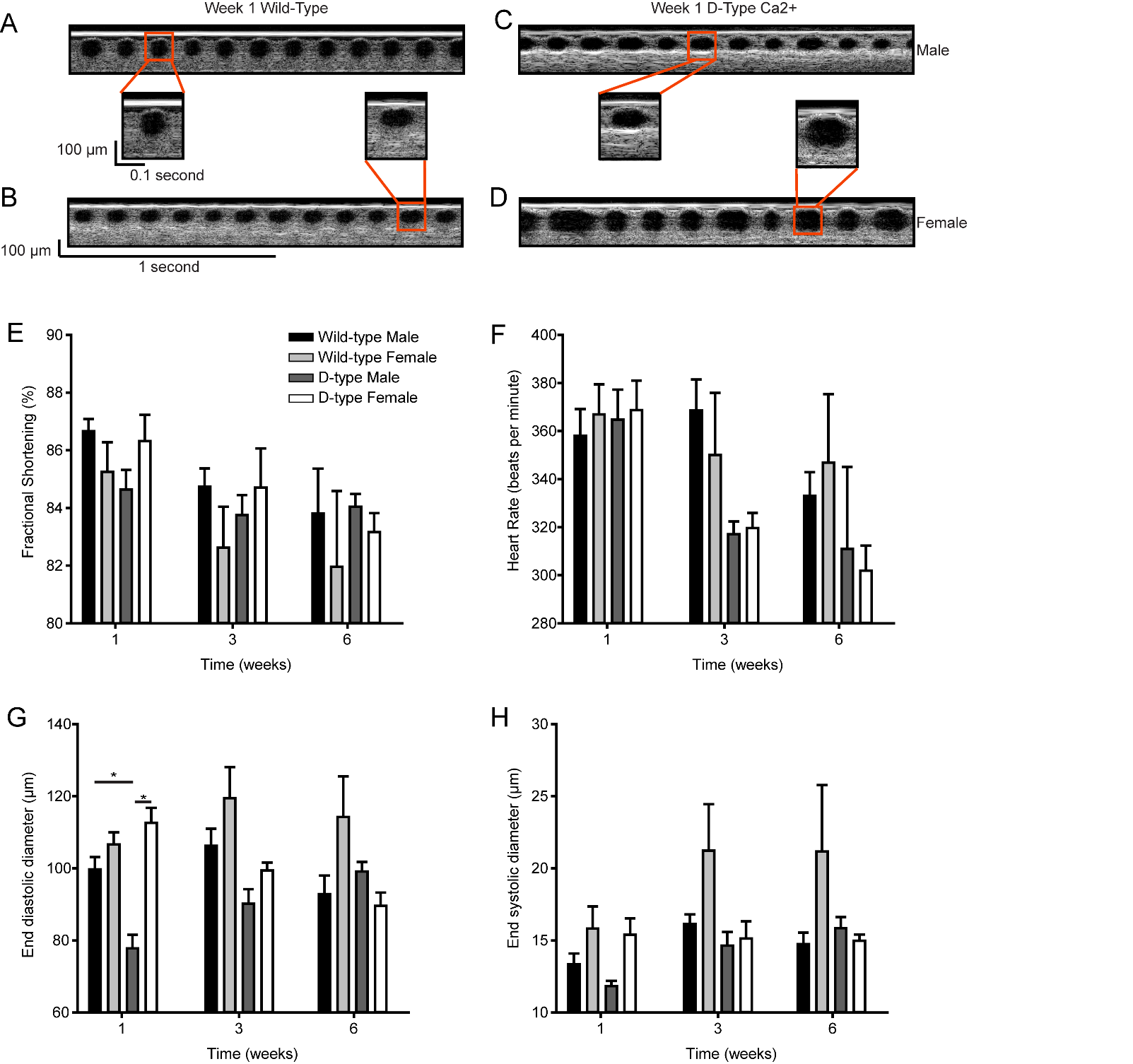

Supplement: Supplementary file 1 — Supplementary Information [file 41598_2018_25195_MOESM1_ESM.docx]
